# Influence of Supercritical Fluid Extraction Process on Techno-Functionality of Enzymatically Derived Peptides from Filter-Pressed Shrimp Waste

**DOI:** 10.3390/md23030122

**Published:** 2025-03-11

**Authors:** Narjes Badfar, Ali Jafarpour, Federico Casanova, Lucas Sales Queiroz, Adane Tilahun Getachew, Charlotte Jacobsen, Flemming Jessen, Nina Gringer

**Affiliations:** 1 Research Group for Bioactives—Analysis and Application, Division of Food Technology, National Food Institute, Technical University of Denmark, DK-2800 Copenhagen, Denmark; naba@bio.aau.dk (N.B.); ajafarpour@upsidefoods.com (A.J.); lusaqu@food.dtu.dk (L.S.Q.); atige@food.dtu.dk (A.T.G.); chja@food.dtu.dk (C.J.); 2 Research Group for Food Production Engineering, Division of Food Technology, National Food Institute, Technical University of Denmark, DK-2800 Copenhagen, Denmark; fjes@food.dtu.dk

**Keywords:** enzymatic hydrolysis, supercritical fluid extraction, interfacial rheology

## Abstract

This study explored how combining supercritical fluid extraction (SFE) and enzymatic hydrolysis influences the structure and functionality of peptides recovered from filter-pressed shrimp waste. Freeze-dried press cake (PC) was defatted via SFE and hydrolyzed using Alcalase (ALC) and trypsin (TRYP). ALC-treated PC achieved the highest protein recovery (63.49%), extraction yield (24.73%), and hydrolysis degree (18.10%) (*p* < 0.05). SFE-treated hydrolysates showed higher zeta potential (−47.23 to −49.93 mV) than non-SFE samples (−25.15 to −38.62 mV) but had larger droplet sizes, indicating lower emulsion stability. SC-ALC displayed reduced fluorescence intensity and a red shift in maximum wavelength. TRYP hydrolysates reduced interfacial tension (20 mN/m), similar to sodium caseinate (Na-Cas, 13 mN/m), but with lesser effects. Dilatational rheology showed TRYP hydrolysates formed stronger, solid-like structures. These results emphasize protease efficacy over SFE for extracting functional compounds, enhancing shrimp waste valorization.

## 1. Introduction

Globally, shrimp production has seen substantial growth, leading to an increased production of side streams that are often discarded or used for low-value products such as mink feed [1]. In 2018, the worldwide accumulative value of wild and aquaculture crustaceans reached 15.38 million MT, with a share of 8% and 52.9% for wild and farmed shrimp species, respectively [2]. However, current predictions put shrimp production at 7.28 million MT in 2025, a significant increase over the 4.57 million MT produced in 2021 [3]. Shrimp is usually processed for meat recovery, while the remaining head and shell portions are side streams [4]. Thus, the value of 50–60% waste of the catch volume from the shrimp processing industry implies the presence of enormous potential for the recovery of a large quantity of valuable compounds, including chitin, lipid, carotenoid, minerals, and especially proteins [5].

To reduce the carbon emissions of the food supply chain, as well as critical environmental and burdensome economic problems, it is necessary to introduce and develop sustainable approaches in both food production systems and waste management [6]. In recent years, considerable attention has been directed towards replacing conventional methods with novel techniques in food science [7]. Conventional methods are typically time-consuming and rely on organic solvents, which results in low extraction yields and undesirable product quality due to the effects of these solvents [7,8]. Therefore, achieving efficient and cost-effective processing of shrimp side streams is crucial for the recovery of high-value biomolecules. Among the different extraction techniques, enzymatic hydrolysis stands out as an environmentally friendly method, offering advantages such as low cost, process repeatability, minimal damage to the nutritional value, and the generation of techno-functional peptides [9]. 

The novelty of the current study is to introduce an efficient cascade extraction approach using green technologies for the recovery of techno-functional peptides from shrimp press cake. The latter was obtained by micro-filter pressing of shrimp heads and shells [10], followed by enzymatic hydrolysis of the proteins, to obtain functional peptides.

The potential functionality of enzymatically recovered peptides can be influenced by factors such as the type of substrate, peptide length, and the amino or carboxyl-terminal amino acids [9]. Additionally, the specificity and selectivity of the enzyme hold a central role in determining the final properties of the peptides [11]. Alcalase and trypsin are recognized as efficient enzymes for generating techno-functional peptides from various sources [12,13]. Hence, in this study, Alcalase and trypsin were selected—due to their high specificity and wide availability [13,14]—to compare their effects on the techno-functionality of the derived hydrolysates from shrimp side streams.

Moreover, the application of a pre-treatment before enzymatic hydrolysis may either facilitate protein recovery and increase efficiency or alter protein conformation with subsequent positive or negative impacts [15]. Therefore, in the present study, the supercritical fluid extraction technique (SFE) was introduced as a pre-treatment to the raw substrate before enzymatic hydrolysis, aimed at recovering astaxanthin-rich oil. Although previous studies have studied the effect of supercritical CO_2_ pre-treatment on the functionality of different biomasses [16], no studies have reported on the effect of supercritical CO_2_ pre-treatment on the structure of peptides obtained from shrimp side streams. Therefore, the objective here was to investigate whether SFE induces structural changes to the peptides obtained from shrimp side streams and whether this would influence their functionality, with a particular focus on the properties of the peptides derived at oil–water interfaces. It was hypothesized that SFE could enhance protein recovery by loosening up the calcified matrix of shrimp waste, thereby providing higher accessibility to the chitin–protein fibers embedded with mineral crystals in the exocuticle of shrimp shells [17]. Therefore, this research has the potential to significantly advance the field of peptide recovery from shrimp waste and aims to explore the potential of SFE as a green and efficient extraction method, emphasizing the importance of comprehending the intricate relationship between the type of processing, molecular structure, and functional properties in the recovery of valuable techno-functional compounds.

This study assessed the mass balance, with respect to protein recovery, the degree of hydrolysis, and the potential for structure modification induced by SFE. The techno-functionality of the recovered peptides was evaluated based on their performance in a fish oil emulsion system, characterized by creaming, zeta potential, and droplet size analysis. Furthermore, the ability of the derived peptides to lower interfacial tension (IFT) at the water-in-oil interface was assessed through the IFT test along with dynamic behavior at the stabilized interface using the dilatational rheology technique and Lissajous plot analysis.

## 2. Results and Discussion

### 2.1. Biochemical Properties of SPHs

Table 1 presents the degree of hydrolysis (DH) of hydrolysates obtained from samples both with and without SFE pre-treatment, total nitrogen content, protein recovery, and yield. In shrimp head and shell powder, protein and chitin represent distinct nitrogen compound sources [18]. The total nitrogen measurement obtained using the Dumas method reveals significant differences in the results, reflecting variations in the chitin–protein mixture across all samples. The highest value was recorded in PC-TRYP (88.60%), while the lowest was observed in SC-ALC (80.89%) (*p* < 0.05). The protein recovery of different hydrolysates was calculated based on amino acid composition. In both pre-treated and non-pre-treated hydrolysates, ALC showed higher protein recovery than TRYP hydrolysates (*p* < 0.05). The DH values obtained may vary depending on experimental conditions such as enzyme type and substrate [19]. In this study, the main variable investigated was enzyme type, which significantly influenced DH. ALC and TRYP yielded DH values ranging from 11.85 to 18.1%. Significant differences were observed among all hydrolysates, including those before (PC) and after SFE pre-treatment (SC) (*p* < 0.05). ALC application in both PC and SC samples at the same concentration (1% *w*/*w* of enzyme/dried matter) resulted in significantly higher DH values compared to TRYP (*p* < 0.05), likely due to differences in enzyme characteristics. ALC, with its specified activity of 2.4 AU/g, cleaves peptide bonds randomly, resulting in broad hydrolysis [20]. Conversely, trypsin with an activity of 6.0 AU/g selectively cleaves peptide bonds at the carboxyl group of arginine or lysine [21] Similar findings were reported by [22] regarding TRYP and ALC application in shrimp by-products. Moreover, the enzymatic hydrolysis in this study was conducted at pH 8.5 to accommodate the high activity for both Alcalase and trypsin. However, [23] reported that trypsin has a narrower optimal pH range, typically between 7.8 and 8.1. While trypsin can still function at pH 8.5, its activity may be slightly reduced compared to its peak efficiency within its optimal range. This could also explain the potential lower efficiency of trypsin observed in the process. Nonetheless, the overall hydrolysis was effective, as Alcalase remained fully active at this pH, ensuring sufficient proteolysis.

Table 2 elaborates on the amino acid composition of each shrimp protein hydrolysate (SPH) and the initial powders (PC and SC). The results show that SC-ALC recorded the highest total amino acid content at 984.12 mg/g, followed closely by SC-TRYP at 968.52 mg/g, whereas PC-TRYP had the lowest content at 833.99 mg/g. These results confirmed that the various types of enzymes affect the amino acid composition differently. ALC facilitates extensive hydrolysis and can cleave most peptide bonds, thereby releasing a larger quantity of amino acids compared to TRYP. In contrast, TRYP predominantly cleaves peptide chains at the carboxyl side of lysin and arginine [24]. In both ALC and TRYP treatments, samples subjected to SFE pre-treatment exhibited higher total amino acid amounts than the non-treated samples. [25] investigated the protein recovery process of squid viscera using the supercritical CO_2_ technique. Their findings indicated a notable increase in the total amino acid content following supercritical extraction. While not explicitly stated in their research, it can be inferred that the supercritical extraction method caused structural changes and protein denaturation. This likely rendered the protein components in the viscera more susceptible to enzymatic hydrolysis, leading to the cleavage of peptide bonds and the formation of smaller peptides and amino acids. Notably, several aromatic hydrophobic amino acids—such as PHE (phenylalanine), ILE (isoleucine), LEU (leucine), TRP (tryptophan), and TYR (tyrosine)—were present in reduced quantities in the SC powder after SFE pre-treatment compared to the original PC powder that had not undergone SFE pre-treatment. This observation may be attributed to the removal of oil from the powder through SFE pre-treatment in the SC powder. Given that the oil was rich in pigments, some may have comprised carteno-proteins [26]. Carteno-proteins represent stable products formed in shrimp shells, where unstable carotenoids link to hydrophobic sites on proteins, thereby enhancing the stability of the carotenoids [27]. Thus, the removal of oil-rich pigments from the powder through SFE could have contributed to a decrease in the concentration of specific amino acids in the initial powder with pigments, as well as in the resulting hydrolysates (SC-ALC and SC-TRYP). Furthermore, the results indicated that, in the comparison between ALC and TRYP treatments, the concentrations of aromatic amino acids in PC-ALC were higher than those observed in PC-TRYP. However, the trend observed in the SC hydrolysates was reversed, with the total concentration of aromatic amino acids in SC-TRYP being higher than that found in SC-ALC. This suggests that, after SFE pre-treatment, ALC’s accessibility to aromatic amino acids decreased. Alternatively, applying SFE as a pre-treatment may induce structural changes in the protein, such as denaturation and unfolding [28]. Consequently, the accessibility of specific amino acids to enzymatic cleavage may be affected. These structural modifications might be a result of variations in the distribution of hydrophobic amino acids within the resulting hydrolysates. However, more research is required to understand the mechanisms underlying these observations. The data show notable differences in the concentrations of hydrophobic and hydrophilic amino acids among various sample treatments. Hydrophobic amino acids, such as tryptophan (TRP), phenylalanine (PHE), leucine (LEU), isoleucine (ILE), methionine (MET), valine (VAL), tyrosine (TYR), alanine (ALA), and proline (PRO), which are characterized by non-polar side chains and are often found in the interior of proteins, away from water, generally exhibited higher concentrations in the untreated press cake (PC) samples compared to the defatted (SC) samples. This is particularly evident in the PC-ALC and PC-TRYP groups.

TRYP samples demonstrate a more hydrophilic character in the peptides produced from these treatments. This change may result from the SFE process, which removes some hydrophobic components along with fats, thereby reducing levels of specific amino acids. Moreover, the high pressure involved in the SFE process can lead to protein denaturation, which makes peptide bonds more readily available for hydrolysis during the defatting process. In contrast, hydrophilic amino acids like threonine (THR), histidine (HYS), lysine (LYS), arginine (ARG), serine (SER), hydroxyproline (HYP), glycine (GLY), glutamate (GLU), aspartate (ASP), and cysteine-cysteine (C-C) have disulfide bonds that feature polar or charged side chains that interact positively with water molecules. These amino acids exhibit varying trends across different treatments. In summary, the differences in hydrophobic and hydrophilic amino acid content indicate that the combined approach of defatting via SFE followed by enzymatic hydrolysis modifies the structure of peptides extracted from shrimp waste. This process likely results in peptides with different functional properties, which could be beneficial for various applications in food and pharmaceutical industries.

### 2.2. FPLC/SEC-MS

**Figure 1:**
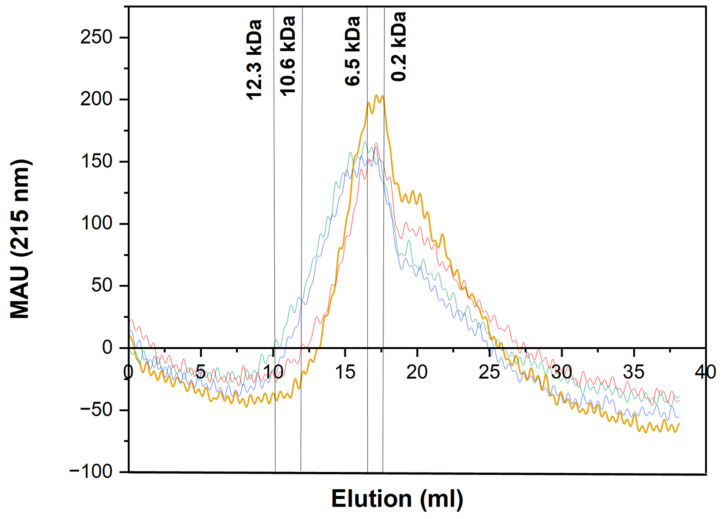
Size exclusion chromatograms illustrating the profiles of shrimp shell hydrolysates (SPHs) obtained without or after supercritical fluid extraction (SFE). (—PC-ALC —SC-ALC, —PC-TRYP —SC-TRYP).

Fast protein liquid chromatography (FPLC) analysis based on size exclusion chromatography was performed on SPHs before and after SFE pre-treatment (Figure 1). The results indicate that both SFE-treated and non-treated hydrolysates exhibited similar trends in their recorded patterns. Additionally, regardless of the type of enzyme applied, both ALC and TRYP produced low-molecular-weight peptides, primarily below 12.3 kDa. Moreover, in TRYP samples (both SC and PC), there was a higher contribution from peptides between 10.6 and 12.3 kDa compared to that from ALC treatments. Conversely, ALC treatments, both in PC and SC samples, displayed higher concentrations of peptides within the 0.2–6.5 kDa range compared to TRYP. This difference may be attributed to the enzymatic specificity, as TRYP cleaves peptide bonds at the C-terminal of lysine and arginine residues, while ALC exhibits a broader catalytic function [29]. Overall, the results suggest that, regardless of SFE pre-treatment, a 2 h enzymatic hydrolysis process could generate short-chain peptides, with SFE having minimal effects on the distribution of peptide molecular weights. The noise in the chromatograph could be attributed to the difference in the conductance between the buffer and the sample, which can lead to slight variations in signal intensity. Similar results of chromatographs have been reported previously [30].

### 2.3. Intrinsic Fluorescence

**Figure 2:**
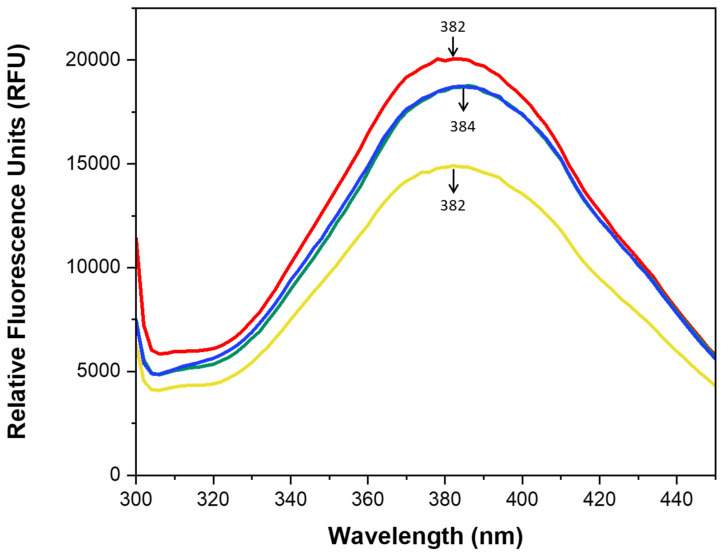
Intrinsic fluorescence of SPHs without and after SFE. (—PC-ALC —SC-ALC, —PC-TRYP —SC-TRYP). The normalized version of Intrinsic fluorescence is available in the supplementary file as Figure S1.

Fluorescence spectroscopy is a commonly used technique for analyzing protein structure and dynamics. It is also employed to monitor proteolysis by measuring changes in intrinsic tryptophan fluorescence [31]. In the present study, the intrinsic fluorescence was measured to investigate the structural modifications in SPHs without and with SFE pre-treatment. The intrinsic fluorescence parameter is primarily attributed to the aromatic amino acids, and the emission spectra depend on the polarity of the molecular environment surrounding aromatic amino acid residues [32]. Enzymatic hydrolysis leads to an increase in exposure of aromatic amino acids (TRP, PHE, and TYR) that are initially hidden in the native protein structure. This process induces conformational alterations, consequently affecting the local environment surrounding the aromatic residues [33]. The results of intrinsic fluorescence and the normalized versions of them are available in Figure 2 and Figure S1. According to Figure 2, the results indicated that both SC and PC hydrolysates exhibited the same maximum fluorescence emission for TRYP (384 nm) and ALC (382 nm) (Figure 2). Tryptophan undergoes excitation at approximately 280 nm and emits fluorescence with a peak intensity of around 332 nm [34,35]. The ALC sample subjected to SFE treatment exhibited a reduction in relative fluorescence units (RFU), indicating an influence on the fluorescence intensity of the sample. This phenomenon could be attributed to alterations in the protein structure, which facilitate the movement of previously buried fluorophores within the protein matrix and result in increased exposure of hydrophobic amino acids [28,36]. A similar result was reported by [16] in egg white protein treated with supercritical CO_2_ fluid extraction at a pressure of 9 MPa for 90 min. On the other hand, the changes observed in the TRYP samples were not substantial, suggesting that, even under SFE treatment, the intrinsic hydrophobicity of the TRYP hydrolysates remained relatively at the same level as that observed for PC-TRYP. TRYP and ALC samples displayed a red shift in comparison with the maximum intensity of tryptophane, which is around 332 nm [34]. However, in terms of the type of enzyme, there was a slight red shift recorded at the maximum wavelength, with a maximum shift of 2 nm. This observation highlights differences in the conformation of the peptides generated by ALC and TRYP. The higher intensity observed for TRYP may be attributed to the increased exposure of tryptophan to the solvent following hydrolysis in these samples. According to [33], the fluorescence spectrum peak in milk protein hydrolysates generated by Alcalase, Flavourzyme, and Protamex exhibited a red shift compared to the non-treated milk protein. Additionally, the fluorescence emission spectra of hydrolysates obtained from Alcalase and Protamex digestion demonstrated substantially lower intensity compared to those treated with Flavourzyme, suggesting that the relative fluorescence intensity may be related to the specificity of each enzyme. The results of intrinsic fluorescence in the current study revealed that SFE pre-treatment induced conformational changes in the ALC samples, but not in the TRYP samples, suggesting that the relative fluorescence intensity in the TRYP samples was influenced solely by enzyme specificity, in contrast to the ALC samples.

### 2.4. Surface Hydrophobicity (H_0_)

Surface hydrophobicity (H_0_) measurements were conducted to assess the behavior of SPHs before and after SFE when dispersed in an aqueous phase (Figure S2). The H_0_ parameter serves as a crucial indicator of conformational and structural alterations within proteins and significantly influences their functional properties, particularly in terms of interfacial behavior and emulsifying properties [28,37,38]. According to [38], the hydrophobicity property negatively correlates with the interfacial tension value, and proteins with higher hydrophobicity values exhibited lower interfacial tension. By dispersing the protein hydrolysates in the aqueous environment, the changes in the secondary structure or conformation of peptides may become apparent [39,40]. According to the results, the surface hydrophobicity of PC-TRYP was significantly higher compared to that of the other hydrolysates (*p* < 0.05), suggesting that TRYP in both SC and PC powders exhibited a higher H_0_ value than the ALC samples when considering enzyme application. This observation may be attributed to the inability of ALC to expose anionic sites, consequently resulting in lower hydrophobicity within the aqueous phase [33]. Similar findings were reported by [33] for Alcalase hydrolysate derived from milk protein after pre-treatment with supercritical CO_2_, wherein the H_0_ value was lower compared to that of Flavoenzyme and Protamex. Additionally, the samples pre-treated with SFE failed to properly position their side chains and side groups in contact with the aqueous phase compared to the PC samples. SFE pre-treatment is suggested to have impacted the protein structure through interactions with alcoholic or amine residues, resulting in the formation of amide bonds. However, according to the results, the SFE process only had an effect on TRYP hydrolysates, and PC-ALC and SC-ALC hydrolysates displayed similar behavior in the aqueous phase (*p* > 0.05). In terms of TRYP hydrolysates, it is postulated that the dissolved supercritical CO_2_ can interact with the protein, leading to irreversible alterations in its structure and conformation [28]. Furthermore, elevating the temperature of the SFE process above 50 °C may decrease the surface hydrophobicity value. This occurrence results from the interaction between supercritical CO_2_ and water, which produces carbonic acid. This carbonic acid then dissociates into bicarbonate, carbonate, and hydrogen ions, reducing the electrostatic forces on the protein molecule’s surface [41]. Ref. [42] also reported a similarly significant reduction in the surface hydrophobicity value following supercritical treatment of egg white protein.

### 2.5. Interfacial Tension and Dilatational Rheology

**Figure 3:**
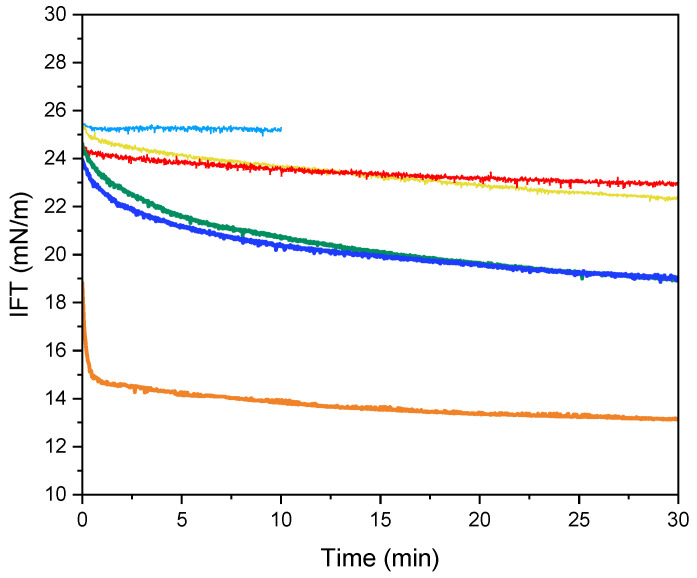
Interfacial tension (IFT) of shrimp shell protein hydrolysates (SPHs). (—PC-ALC —SC-ALC, —PC-TRYP —SC-TRYP), sodium caseinate (—Na-Cas) as positive control and (—W/O) water in oil droplet.

Due to their amphiphilic nature, proteins and peptides can form a viscoelastic network at interfaces [43]. This study evaluated the influence of 0.1 wt% SPHs, with and without SFE pre-treatment, along with Na-Cas (positive control) and water (negative control), on interfacial tension (IFT) reduction over time (Figure 3). The IFT between water droplets and MCT oil remained constant at 25 mN/m, indicating the absence of surface-active compounds in the water droplets. Similar IFT values for water droplets have been reported previously [43,44]. Na-Cas exhibited a rapid decrease in IFT to 15 mN/m within the first 2 minutes, followed by a further reduction to 13 mN/m at 30 min. This initial sharp decline can be attributed to protein adsorption at the interface, while the subsequent gradual decrease reflects protein conformational rearrangement [45]. Previous studies have similarly demonstrated the effectiveness of Na-Cas in reducing IFT at water–oil interfaces [44,46]. In contrast, ALC hydrolysates, with and without SFE pre-treatment, showed minimal changes in IFT and exhibited higher initial and equilibrium interfacial tension values compared to TRYP hydrolysates. The interfacial properties of peptide and protein adsorption are influenced by factors such as charge, concentration, conformation, and surface hydrophobicity [47]. TRYP hydrolysates, both with and without SFE pre-treatment, demonstrated consistent reductions in IFT over 30 min, reaching a minimum value of 19 mN/m. This consistent reduction, resembling that of Na-Cas, but with a less pronounced effect, aligns with previous findings. Reference [48] reported that larger potato peptide fractions generated by trypsin (>10 kDa) exhibited higher IFT reduction compared to smaller peptides and unfractionated potato hydrolysates. This suggested that the amphiphilic properties varied among the peptide fractions [20]. Surface hydrophobicity results (H_0_ value) were consistent with the IFT findings, showing higher values for TRYP hydrolysates compared to ALC samples. Additionally, samples unaffected by SFE displayed lower IFT values compared to those subjected to SFE.

**Figure 4:**
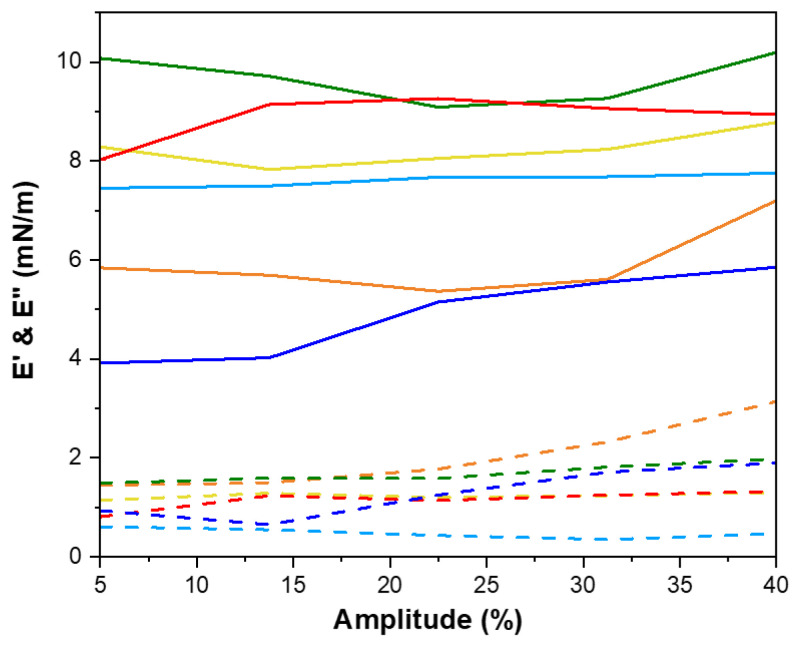
Amplitude sweep test of shrimp shell hydrolysates (SPHs) and controls. (—PC-ALC —SC-ALC —PC-TRYP —SC-TRYP), sodium caseinate (—Na-Cas) as positive control and (—W/O) water in oil droplet. Elastic modulus (E′) is represented with symbol line and viscous modulus (E″) is represented with dotted line.

**Figure 5:**
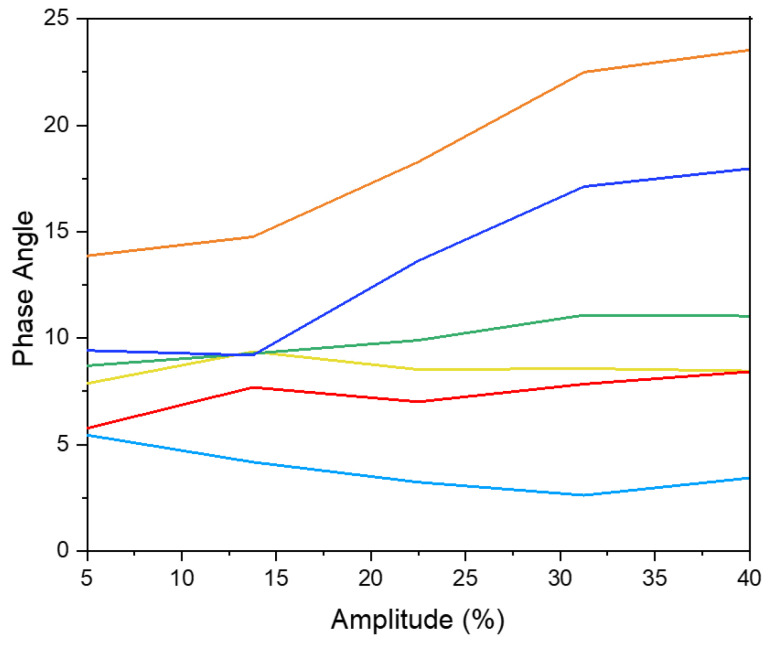
Phase angle of shrimp shell hydrolysates (SPHs) and controls. (—PC-ALC —SC-ALC, —PC-TRYP —SC-TRYP), sodium caseinate (—Na-Cas) as positive control and (—W/O) water in oil droplet.

**Figure 6:**
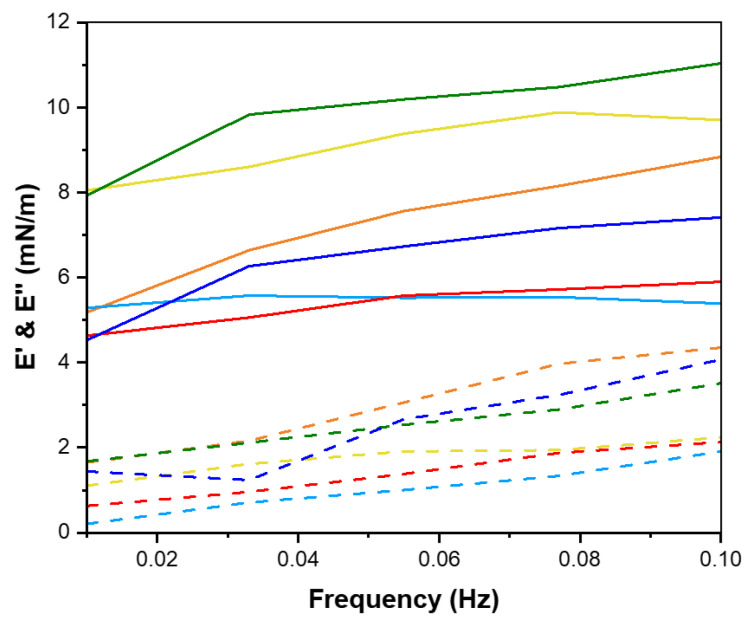
Frequency sweep test of shrimp shell hydrolysates (SPHs). (—PC-ALC —SC-ALC, —PC-TRYP —SC-TRYP), sodium caseinate (—Na-Cas) as positive control and (—W/O) water in oil droplet. Elastic modulus (E′) is represented with symbol line and viscous modulus (E″) is represented with dotted line.

In summary, the capacity of peptides to reduce interfacial tension is linked with their surface hydrophobicity, underscoring the importance of peptide structure in interfacial activity [47]. The amphiphilic nature of proteins and peptides enables them to act as effective emulsifiers at the interface, thereby stabilizing the interfacial layer. This emulsifying capacity is closely linked to interfacial rheological properties, specifically dilatational rheology, which reflects the organization of peptides at the interface [49]. Notably, no studies have reported on the interfacial dilatational rheology of shrimp head and shell hydrolysates or examined the effects of supercritical fluid extraction at the water-in-oil (W/O) interface. In the context of dilatational rheology, amplitude and frequency oscillation responses are highly significant. The storage modulus (E′), representing the stored energy during relaxation processes, is a key parameter elucidated by dilatational modules [50]. Amplitude sweep analysis is crucial for understanding the strength and viscoelastic response of the interfacial layer in relation to deformation amplitude [51]. Under a constant frequency of 0.01 Hz, amplitude sweep tests were conducted on Na-Cas, hydrolysates, and water droplets to identify linear viscoelastic regions across different amplitude ranges (Figure 4). The response of the water droplet in the oil phase remained constant within the deformation range of 5% to 40%, attributed to the absence of surface-active compounds. Conversely, Na-Cas, as a positive control, exhibited an increase in storage modulus (E′), indicating a viscoelastic response to amplitude changes. Similar results were reported by [43,52]. Remarkably, both PC-ALC and SC-ALC demonstrated behavior resembling that of the water droplet as the amplitude increased from 5% to 40%. Furthermore, both PC-TRYP and SC-TRYP exhibited a viscoelastic response, with SC-TRYP showing the highest E′, indicative of enhanced intermolecular interactions that improve interface elasticity and mechanical strength [48]. Conversely, the lowest E′ was observed in PC-TRYP, indicating a weaker but highly stretchable interface. The storage modulus in the SC-TRYP samples was higher than that of the Na-Cas samples, with noticeable alterations observed with increasing amplitude. Notably, the Na-Cas interface exhibited weakness but high stretchability, consistent with findings by [51]. However, hydrolysates formed a rigid and brittle interface, as further discussed in the subsequent sections. The phase angle is defined as a parameter reflecting changes in viscosity and elasticity, calculated based on the elastic and viscous modules. Values between 0 and 90 indicate viscoelastic behavior, with perfectly elastic and viscose material having phase angle values of 0 and 90, respectively [53]. Notably, an increase in amplitude value resulted in a decline in the phase angle for the water sample (Figure 5). This observation suggests that the behavior of water at the interface led to increased resistance and exhibited solid-like characteristics. In contrast, for the Na-Cas droplet, the phase angle increased with rising amplitude values, indicating viscous behavior. Additionally, there were no changes in the phase angle for PC-ALC and SC-ALC, suggesting that these samples lacked viscoelasticity, which could be due to the low molecular interactions at their interfaces [54]. On the other hand, PC-TRYP and SC-TRYP exhibited a pattern similar to Na-Cas, but with lower viscoelasticity. The combination of larger dilatational moduli and a lower phase angle in these samples suggests the formation of a stronger interfacial layer [55]. Frequency sweep analysis was conducted in the range of 0.01 to 0.1 Hz with a fixed amplitude of 5% (Figure 6). It should be noted that frequencies exceeding 0.1 Hz may induce droplet deflection at the interface [56]. The response of the samples to changes in frequency was similar to that observed during amplitude sweep analysis. Specifically, an increase in frequency corresponded to an increase in storage modulus (E′). All samples exhibited frequency-dependent behavior, with consistently higher storage moduli (E′) recorded compared to loss moduli (E′′). [55] reported that interfacial layers stabilized by enzymatic hydrolysates from whey protein displayed constant and frequency-independent storage moduli. In contrast, within the examined frequency range in the present study, both E′ and E′′ in hydrolysates and Na-Cas increased, indicating a viscoelastic response. SC hydrolysates (SC-ALC and SC-TRYP) showed the highest elasticity at the oil–water interface, outperforming both Na-Cas and PC hydrolysates. Among the Na-Cas and PC hydrolysates, Na-Cas exhibited higher elasticity than PC hydrolysates. This suggests that Na-Cas has better stretchability compared to PC hydrolysates. According to [55], samples with higher E′ values were characterized as being more rigid, while those with lower E′ values displayed greater stretchability. The storage modulus reflects the strength of intermolecular linkages formed by conformational changes and interactions between proteins and peptides. These linkages contribute to the resistance of deformation at the interface [57].

### 2.6. Lissajous Plots

**Figure 7:**
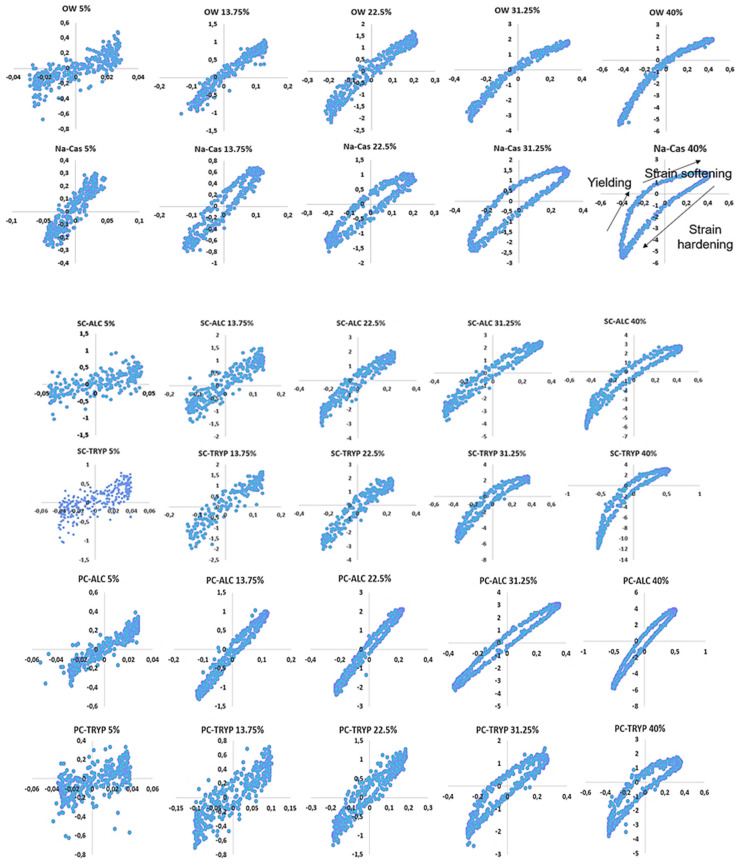
Lissajous plots of 0.1% SPHs, Na-CAS solution (positive control), and water-in-oil droplet (W/O) under 5, 13.75, 22.5, 31.25, and 40% amplitude.

The impact of shrimp enzymatically derived peptides, particularly those influenced by SFE, has not been explored using the Lissajous plots method before. To further elucidate the rheological characteristics of the interface, Lissajous plots were employed. This analysis is defined as a strong tool, particularly in the nonlinear regime, offering valuable insights into the viscoelasticity, molecular, and microstructural behavior of the interfacial layer [58]. The shape of this plot gives direct indications regarding the behavior of the interfacial film in dilatational deformation. A straight line, circle, or elliptic shape corresponds to an elastic, viscous, or viscoelastic response, respectively [51]. In addition, the nonlinear behavior of the oil–water interface results in asymmetric Lissajous plots, for instance, asymmetry may reveal softening in the expansion (the upper right part of the plot) or hardening in compression (the lower left part of the plot) [51]. As depicted in Figure 7, the water droplet straight pattern (closed plot) indicates no significant response to increasing the amplitude. In contrast, the Na-Cas droplet demonstrated a distinct response as the amplitude increased from 5% to 13%, resulting in the opening and formation of an elliptical shape in the Lissajous plots. This behavior indicates the emulsifying properties of the protein and the stability of the interface. However, at amplitudes exceeding 22.5%, the elliptical shape exhibited slight asymmetry, suggesting a destabilization of the Na-Cas interface. This instability becomes more recognizable at 40% amplitude. Furthermore, at this point, protein bonds began to break, leading to the rupture of the interfacial network—a phenomenon known as strain softening [47]. In the case of PC-ALC, the response to the varying amplitudes closely resembled that of water, with minimal deviation in the Lissajous plots and a predominantly linear elastic response to increasing amplitude. This observation suggests that PC-ALC peptides exhibited poor emulsifying properties, resulting in the formation of a rigid interface. The Lissajous plot observed in SC-ALC exhibits similarities to the PC-ALC. In both PC and SC samples, the Lissajous plots for TRYP display a greater expansion compared to the ALC samples, indicating a stronger viscoelastic response at the interface. However, the application of SFE pre-treatment led to a slight modification of peptides, resulting in a greater widening of the ellipse on the Lissajous plots compared to the samples without SFE pre-treatment. This observation suggests that SFE pre-treatment has the potential to alter peptide conformation, albeit not entirely negatively.

### 2.7. ζ-Potential, Droplet Size, and Creaming Index of Oil-in-Water Emulsions

The results of ζ-potential and droplet size for SPHs emulsions and Na-Cas emulsion are presented in Table 3. The key factors contributing to emulsion stability include droplet size, surface charge, and interfacial free energy. The ζ-potential is a parameter that provides insights into both potential emulsion stability and surface charge at the oil–water interface. Essentially, this parameter elucidates the particles’ capacity to attract each other at the interface layer [44]. In the present study, the ζ-potential of emulsions stabilized with 0.2% SPHs or Na-Cas ranged from −49.93 mV to −25.15 mV. Significant differences were observed between the emulsions (*p* < 0.05). A higher absolute value of the ζ-potential indicates increased electrostatic repulsion among the emulsion droplets and subsequently contributes to increased emulsion stability [59]. However, the nature and source of the protein and peptides play an important role, and, despite the high absolute value of ζ-potential, the resulting emulsion might not be stable [60]. Na-Cas, as a positive control, recorded a value of −43.77 mV. A similar result was reported by various studies, such as [44,61,62]. A ζ-potential above ׀25-30׀ mV suggests enhanced emulsion physical stability, indicating that Na-Cas facilitated strong electrostatic repulsion between droplets. Furthermore, the recorded values for SC-ALC, SC-TRYP, and PC-TRYP did not exhibit significant differences compared to Na-Cas (*p* > 0.05). On the other hand, the PC-ALC sample exhibited the lowest ζ-potential, indicating potentially reduced emulsion stability compared to the other treatments (*p* < 0.05).

**Figure 8:**
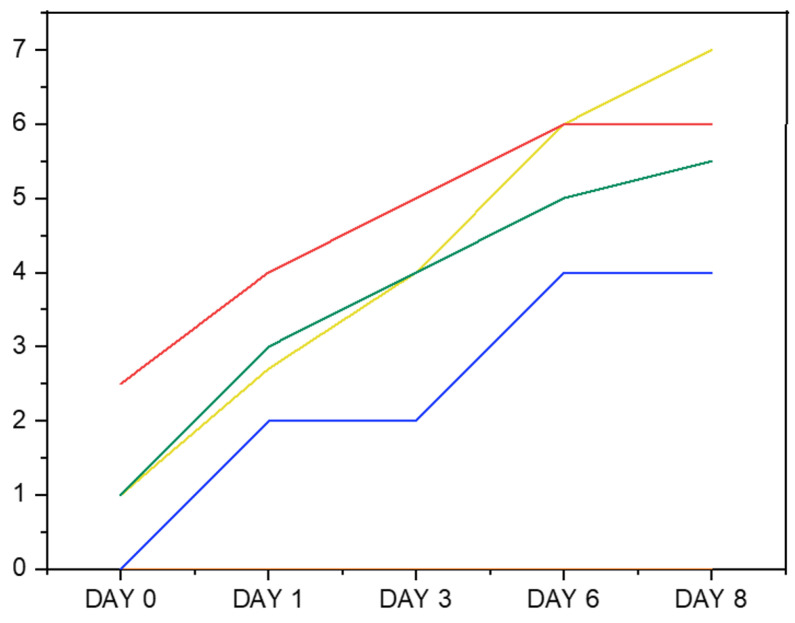
Creaming index of emulsions stabilized with 0.2% protein of SPHs and Na-Cas. (—PC-ALC —SC-ALC, —PC-TRYP —SC-TRYP —Na-Cas).

The droplet size of emulsions stabilized by two concentrations of 0.2 and 0.4% SPH solutions were measured and compared to the droplet size of Na-Cas emulsion as a positive control (Table 3). The droplet sizes of emulsions stabilized by SPHs were initially measured at a hydrolysate content concentration of 0.2%. The results revealed that, at this concentration, the emulsion yielded large droplet sizes (D4,3) exceeding 23–67 µm on day 1 and 28–71 µm on day 8. This observation led us to hypothesize that the peptide content may have been insufficient at the oil–water interface. To investigate whether increasing the peptide concentration would lead to smaller droplets, we repeated the experiment using a higher peptide concentration of 0.4%. Even when increasing the protein/peptide content from 0.2 to 0.4%, the emulsions produced by the SPH solution exhibited large-sized droplets. This observation suggests that, while increasing the concentration of SPHs facilitates the formation of smaller droplets by providing more protein/peptide molecules to cover the emulsion droplet surfaces, the ability of SPHs to stabilize the 5% fish oil in water emulsion remains low compared to Na-Cas. This suggests that the peptides may not exhibit significant surface activity. It is possible that a considerable portion of the peptides are too small and thus remain predominantly in the aqueous phase rather than at the oil–water interface. This could indicate a lack of proper amphiphilic properties among the peptides [46]. Similar findings were reported by [63], proposing that the observed increase in droplet sizes may be attributed to emulsion coalescence, a phenomenon likely influenced by the diminished absorption rate of emulsifiers, resulting from their low concentration. In contrast, Na-Cas at a 0.2% concentration demonstrated low values of D3,2 and D4,3 on the first day (day 1) and last day (day 8) of the storage period. A similar result for caseinate was reported by [44]. The results of droplet size measurement were in line with the creaming index of 0.2% protein hydrolysate visual estimation (Figure 8), indicating a more stable emulsion with the smallest droplet size recorded by Na-Cas during the 8-day storage period. This suggests that an ample quantity of protein was absorbed at the water–oil interface, effectively preventing coalescence, and that the initial droplet size remained sufficiently small enough to prevent physical destabilization of the emulsion [64]. In contrast, the highest creaming index (%) was measured in SC-ALC and PC-ALC. Followed by Na-Cas, the highest emulsion stability among the other SPHs was obtained by PC-TRYP (*p* < 0.05). Furthermore, emulsions treated with TRYP demonstrated greater stability compared to those treated with ALC hydrolysates. Moreover, it was observed that the emulsion stabilized with PC-ALC showed a rapid phase separation from day 0 to day 8 of storage, which could be attributed to its lower net zeta potential. Creaming is a usual observation that occurs in normal polydisperse emulsions [65]. This phenomenon increases with coalescence and flocculation [66].

Overall, the results confirm that, although higher electrostatic repulsion contributes to the physical stability of emulsions, some other factors, such as droplet size, potentially influence the overall physical stability of an emulsion, which is influenced by multi-factors. Moreover, the results of the physical stability test have confirmed the result of interfacial tension analysis.

The results of emulsion physical stability, which show that TRYP emulsions had significantly smaller droplets than ALC emulsions (D3,2) and a lower degree of creaming, correlate with findings from interfacial properties and Lissajous plots. These plots demonstrated a stronger viscoelastic response at the interface for TRYP compared to ALC samples. The different effects of SFE pre-treatment on the emulsion stability of ALC and TRYP hydrolysates, despite using the same SFE pre-treated powder and emulsion production technique, are intriguing and require further investigation. One possible explanation could be the distinct surface-active properties and emulsifying abilities of the peptides generated from ALC and TRYP hydrolysis. Differences in peptide composition and structure may have resulted in varied interactions with the oil–water interface, thereby influencing emulsion stability differently. Additionally, the emulsion stabilization mechanisms may exhibit enzyme-specific effects, leading to contrasting responses to SFE treatment. Further studies examining the specific characteristics of shrimp head and shell peptides generated from ALC and TRYP hydrolysis, as well as their interactions with the emulsion components, are needed to fully understand the underlying factors contributing to the observed differences in emulsion stability.

## 3. Materials and Methods

Shrimp (*Pandalus borealis*) were supplied frozen by (Launis A/S, Skagen, Denmark). After thawing the frozen shrimp, they were transferred into the filter press (PCT/EP2021/068371; ref.: P5654PC00) with a mesh size of 300 μm [9]. The shrimp press cake (PC) was then collected, labeled, and frozen at −20 °C, transferred in insulated boxes on ice from the company to the Technical University of Denmark, and kept at −40 °C. PC was lyophilized with a freeze-drier (Merck, Beta 1–8, Martin Christ^®^ GmbH, Darmstadt, Germany) at −50 °C and pulverized with an electric mill to the size of <250 μm. Distilled deionized water was employed for the preparation of all solutions during hydrolysate production. As a reference for emulsification experiments, sodium caseinate (Na-Cas) (Miprodan 30) was supplied by (Arla Foods Ingredients AmbA, Viby J, Denmark). All chemicals used were of analytical grade. The extraction of proteins was performed with Alcalase 2.4L (2.4 AU/g) and trypsin (Pancreatic Trypsin Novo (PTN) 6.0S (6.0 AU/g), which were provided by Novonesis (formerly known as Novozymes A/S) (Bagsværd, Denmark).

### 3.1. Supercritical CO_2_ Extraction

The supercritical CO_2_ fluid extraction process was applied to the PC powder using a supercritical fluid extraction instrument (MV-10 ASFE System, Waters, Milford, MA, USA). The procedure involved packing approximately 8 g of dried PC powder with less than 1 mm particle size into 25 mL extraction vessels, which were subsequently connected to both a supercritical fluid inlet and an extract outlet line. Then, the vessels were systematically positioned within a temperature-controlled oven. The procedure ensured the initiation and maintenance of the extraction process under environmental conditions. The process was initiated with the introduction of CO_2_ into the extraction vessel to reach the targeted pressure level using a high-pressure pump following the cooling process with the aid of passing CO_2_ through a heat exchanger. The extraction was carried out under pressure of 250 bar, temperature of 45 °C, CO_2_ flow rate of 7 mL/min, and 1 and 3 mL/min cosolvent (ethanol 96% (*v*/*v*)) flow rates. The total duration of the process was 60 min. During the initial 50 min of the dynamic extraction phase, cosolvent was introduced to the system. Subsequently, for the final 10 min of extraction, the flow of cosolvent was stopped, while maintaining the CO_2_ flow rate, aiming to remove any residual solvents from the extraction vessels. Then, the system underwent depressurization, and both the extract and residues were kept for further analysis. A stream of nitrogen was used to remove the remaining solvent in the extract. The yield of extraction was calculated using the following formula:

(1) $$\mathrm{Extraction}\;\mathrm{yield}\;\left(\%\right)=\frac{\mathrm{Weight}\;\mathrm{of}\;\mathrm{the}\;\mathrm{dried}\;\mathrm{extract}}{\mathrm{Weight}\;\mathrm{of}\;\mathrm{shrimp}\;\mathrm{shell}\;\mathrm{powder}}\times 100$$

### 3.2. Enzymatic Hydrolysis and Degree of Hydrolysis

Shrimp protein hydrolysates (SPHs) were produced from lyophilized powders with and without SFE pre-treatment by the application of Alcalase (ALC) and trypsin (TRYP). The samples were dispersed in distilled water (1:5 *w*/*v*) and heated up to 55 °C for ALC and 40 °C for TRYP. After adjusting the pH at 8.5 with 1M NaOH, the hydrolysis reaction was carried out in an enzyme/substrate ratio (E/S) of 0.5% in a shaking water bath with temperature control. Hydrolysis was continued for 2 h. The hydrolysis reaction was carried out under an initial pH adjustment approach without a pH-stat system. The pH was monitored throughout the process, and it remained within both the active and optimal ranges for Alcalase and trypsin, ensuring effective enzymatic hydrolysis. After hydrolysis, the pH was set to 7.0 with 1 M NaOH to ensure consistency in subsequent techno-functional property assessments, as many food applications operate near neutral pH. Then, the sample was heated to 90 °C for 15 min to deactivate the enzyme. To measure the degree of hydrolysis (DH), the liquid part was centrifuged at 3750× *g* at 22 °C for 10 min, and DH was determined from the supernatant with o-phthaldialdehyde (OPA assay), as described by [67]. Afterward, the supernatant samples were freeze-dried to calculate the weight of protein biomass. The protein content was determined using Dumas to calculate the percentage of protein recovery using a conversion factor of 6.25. The SPHs were lyophilized and stored at 4 °C until further analysis.

### 3.3. Protein Content (Based on Total Nitrogen Content), Total Amino Acid Composition, and Protein Recovery % (PRP)

The total nitrogen content was determined using a Dumas instrument (Rapid MAX N exceed cube N/protein analyzer, Elemental Analyzer system GmbH, Frankfurt, Germany). A total of 300 mg of the sample was applied for measurement. The values of the total nitrogen content (%) were multiplied by 6.25 to find the protein content (*n* = 3). The amino acid content was obtained using HPLC-MS, following hydrolysis and derivatization using an EZ:faast amino acid kit (Phenomenex, Torrance, CA, USA), as reported by [10]. Briefly, the acid hydrolysis was applied to release the amino acid using 6 M HCl for 1 h at 110 °C in a microwave sample preparation system (Multiwave 3000, Anton Paar, Graz, Austria). Then, the samples were purified with a solid-phase extraction sorbent tip, and derivatization was carried out following the injection of sample aliquots into an Agilent HPLC 1100 instrument (Santa Clara, CA, USA) linked to an Agilent ion trap mass spectrometer. The amino acids were recognized by comparing the retention time and mass spectrometric profiles of an external reference standard mixture. Calibration curves were generated and analyzed using HPLC-MS for quantification. All analyses were performed in triplicate samples (with two analytical replicates; *n* = 3 × 2).

The total amino acid profile was measured using the acid hydrolysis method, as explained above, and the PRP was calculated using the following formula:

(2) $$PRP\;\left(\%\right)=\frac{fds\;\left(g\right)\times AA_{fds}\left(\%\right)}{AA_{spc}\;\left(g\right)}$$

where fds is the net weight (g) of the freeze-dried sample, $AA_{fds}$ is the sum of amino acid content of the freeze-dried sample, and $AA_{spc}$ is the sum of amino acid content (g) in the raw material (lyophilized PC powders) before enzymatic hydrolyzation.

### 3.4. Fast Protein Liquid Chromatography (FPLC)

FPLC was carried out to characterize the molecular weights of small proteins and peptides. A total of 100 mg of SPHs was extracted in water (2 mL) using a homogenizer (Polytron PT 1200, Kinematica, MA, USA) for 30 s. Then, this was incubated at RT for 30 min, followed by a second round of homogenization (30 s), followed by incubation for 15 min at room temperature, and was then centrifuged for 15 min at 20 °C at 20,000× *g*. The supernatant was filtered (0.2 μm) before starting the analysis with fast-performance liquid chromatography (FPLC) equipment (Akta Purifier system with Frac 950 collector, GE Healthcare Life Sciences, Alderley Park, UK). A total of 100 μL of the sample (corresponding to 5 mg hydrolysates) was injected onto a SuperdexTM peptide 10/300 GL column (GE Healthcare) using 100 mM ammonium acetate, with pH 8 as a running buffer, at a flow rate of 0.25 mL min^−1^. To assess the molecular weight distribution of the generated peptides, the following internal standards were used: Cytochrome c (CytC, 12.3 kDa), bradykinin (10.6 kDa), aprotinin (6.5 kDa), and triglycine (Gly3, 185 Da). Generally, the largest molecule elutes first came from the column. mAU, representing milli absorbance units, correlates with higher concentrations of low-molecular-weight compounds absorbing at 215 nm [30].

### 3.5. Intrinsic Fluorescence Emission Spectroscopy

The intrinsic fluorescence of SPHs was assessed using a fluorescence spectrophotometer (SPECTRAmax GEMINI; Molecular Devices, San Diego, CA, USA). A stock solution was prepared at a concentration of 10 (mg/mL) in phosphate buffer solution (0.1 M, pH = 7). Then, the stock solution was diluted 100-fold to achieve 0.1 mg/mL. The extinction wavelength was set at 280 nm. Subsequently, the emission spectrum was recorded with a scanning speed of 10 nm/s within the range of 300–450 nm, with both extinction and emission measurements conducted under the constant 2 nm slit width. Phosphate buffer was employed as the blank solution for baseline correction.

### 3.6. Surface Hydrophobicity (H_0_)

The surface hydrophobicity (*H*_0_) of the SPHs was assessed using the anionic fluorescence probe 1-anililo-naphthalene-8-sulfonate (ANS). Various concentrations of SPH solutions were used (2.5, 5, 7.5, and 10 mg/mL), which were prepared in 0.01 M phosphate buffer (pH 7.0). Subsequently, a fresh stock of ANS solution (8 mM) was prepared. An aliquot of 20 μL of ANS was then added to each SPH solution, followed by incubation in the darkness for 10 min to ensure proper interaction between the prob and the SPH solution. The fluorescent intensity of the samples was assessed using a 96-well microplate reader (SPECTRAmax GEMINI; Molecular Devices, San Diego, CA, USA) with an extension of 370 nm and an emission of 470 nm. The *H*_0_ value was derived by analyzing the initial gradient of the linear regression plot correlating relative fluorescence intensity (RFI) with protein concentration (%).

### 3.7. Interfacial Tension and Dilatational Rheology

Both tests for the interfacial tension between oil and water and dilatational rheology tests were carried out with a drop tensiometer (OCA20, DataPhysics Instruments, Filderstadt, Baden-Württemberg, Germany) equipped with an oscillating drop generator (ODG20, Dataphysics). Briefly, a volume of 15 μL at 0.01% SPHs or Na-Cas (*w*/*v*) was gently shaped at the tip of a stainless-steel needle (d = 0.51 mm) into the MCT (medium-chain triglycerides) oil phase within a quartz-glass cuvette. The interfacial tension measurements were conducted for 30 min at room temperature to allow adsorption to proceed until reaching the near-equilibrium state. The boundaries of the obtained image of the droplet were digitized with a CCD camera to calculate the interfacial tension (γ) based on the Young–Laplace equation. With a constant droplet volume, the adsorption kinetics to the interface were recorded and reported as IFT (interfacial tension) (mN/m). In terms of the dilatational rheology test, after an equilibration time of 3 h, the linear viscoelastic regime was determined. The deformation amplitude was varied manually (5%, 13.75%, 22.5%, 31.25%, and 40% interfacial area) at a frequency of 0.01 Hz, where each oscillation consisted of 5 cycles, and, before each step, the interfacial film was allowed to recover for 90 s. The frequency sweeps were performed after the amplitude sweeps, and the frequency varied from 0.01 to 0.1 Hz. However, to run the frequency sweep test, the deformation amplitude needs to be within the linear viscoelastic regime. Thus, an amplitude of 5% was chosen in our study, as it provided a linear interfacial tension response at the applied strain. Between each frequency step, the droplet was allowed to rest for 90 s to regain equilibrium. The surface dilatational elastic and viscous moduli ($E_{d}^{\prime }\;$and $E_{d}^{\prime \prime }$, respectively) were determined from the measured dynamic interfacial tension response, obtaining the intensity and phase of the first harmonic after Fourier transformation of the γ signal using the following equations [20]:

(3) $$E^{\prime }=\Delta_{\gamma }\frac{A_{0}}{\Delta A}\mathrm{Cos}\;\emptyset$$

(4) $$E^{\prime \prime }=\Delta_{\gamma }\frac{A_{0}}{\Delta A}\mathrm{Sin}\;\emptyset$$

where Δγ is interfacial tension variation, ΔA is the amplitude of the periodic interfacial area, $A_{0}$ represents the unperturbed interfacial area, and ∅ is the phase angle. Moreover, the response was visualized directly in so-called Lissajous plots, as follows: surface pressure (stress *π* = *γ*_0_ − *γ*) with $\gamma_{0}$ of the interfacial tension before deformation against deformation (strain $\Delta A⁄A_{0}=\left(A_{0}-A\right)/A_{0}$, where $A_{0}\;$is the interfacial area before deformation and A is the interfacial area during oscillation) [51]. Plots were drawn for each deformation from the middle three oscillations.

### 3.8. Emulsion Preparation and Storage Study

The stability of the emulsion was investigated for the SPHs. Accordingly, sodium caseinate (Na-Cas) was utilized to produce a positive control emulsion. Emulsifiers, both SPHs and Na-Cas, were dissolved in distilled water, and the pH was adjusted to 7 using 0.1 M HCl. Emulsions containing 0.2 and 0.4% (*w*/*w*) protein or protein hydrolysate and 5% (*w*/*w*) cod liver oil (from Vesteraalen, Sortland, Norway) were prepared. Initially, a pre-emulsion was formed using an Ultra Turrax (IKA Werke GmbH &. Co., Staufen, Germany) at 16,000 ×g for 2 min. The addition of oil to the aqueous phase was carried out gradually during the first minute of homogenization. Subsequently, homogenization was performed using a high-pressure homogenizer (Panda Plus 2000, GEA Niro Soavi, Lübeck, Germany) at a pressure of 9 Pas with 3 passes. The temperature during the homogenization process was controlled and maintained below 26 °C. The prepared emulsions were then stored in the dark at 20 °C in 10 mL glass bottles for 8 days.

### 3.9. Determination of the Ζ-Potential, Particle Size, and Creaming Index (%)

The ζ-potential of the 0.2% SPHs or Na-Cas emulsions was measured after one day of storage using a Zetasizer Nano ZS instrument (Malvern Instruments Ltd., Worcestershire, UK) equipped with a DTS1070 cell operating at 25 °C. Before analysis, the emulsions were appropriately diluted (20 μL emulsion in 10 mL imidazole buffer 10 mM). The zeta potential range was adjusted to −100 to +50 mV, and each sample underwent analysis with 100 runs. The measurements were conducted in triplicate.

Regarding droplet size and creaming, the droplet size distribution of both 0.2 and 0.4% emulsions was determined via laser diffraction using a Mastersizer 2000 (Malvern Instruments, Ltd., Worcestershire, UK) on days 1 and 8 of storage at room temperature (~22 °C) in dark conditions. Each emulsion was diluted in recirculating water at 3000 rpm until an obscuration of 12% was achieved. The results were reported in terms of volume mean diameter (D4,3 and D3,2). The measurements were performed in triplicate. Creaming of 0.2% protein or protein hydrolysate emulsion was evaluated qualitatively by a visual inspection of the emulsion’s appearance.

### 3.10. Statistical Analysis

All interfacial tension and rheology experiments were performed twice as independent replicates. Analysis of variance (ANOVA) was conducted with the aid of the statistical software (Statgraphics Technologies, Version 18.0.6, Inc., The Plains, VA, USA). For significant ANOVA outcomes, means were compared pairwise (*p* < 0.05) using post hoc Tukey HSD. From the oscillation experiments, the middle 3 cycles per amplitude were used for the analysis of the rheological moduli.

## 4. Conclusions

This study underscores the potential of enzymatic hydrolysis for obtaining functional protein hydrolysates from shrimp by-products. The results indicate that enzyme selection and SFE pre-treatment significantly impact protein characteristics. Specifically, Alcalase (ALC) showed higher hydrolysis efficiency compared to trypsin (TRYP), leading to increased amino acid recovery, especially with SFE pre-treatment. Structural changes induced by enzymatic hydrolysis and SFE pre-treatment were evident through intrinsic fluorescence analysis, affecting protein structure and surface hydrophobicity. TRYP hydrolysates had better interfacial properties. Peptide composition influenced viscoelastic responses, with ALC-based hydrolysates resembling water droplets and TRYP-based hydrolysates showing viscoelastic behavior. These findings contribute to our understanding of the techno-functional properties of enzymatically derived peptides and their potential applications in food industries.

## Figures and Tables

**Table 1:** Results of degree of hydrolysis (DH) (%), total nitrogen content (%), protein recovery (%), and yield efficiency (%).

Sample	DH (%)	Protein Content (%) (Based on Total Nitrogen Content (%))	Protein Recovery (%)	Yield (%)
PC-ALC	18.10 ± 0.14 ^a^	85.69 ± 0.62 ^b^	63.49 ± 0.41 ^a^	24.73 ± 0.70 ^a^
PC-TRYP	14.50 ± 1.27 ^a,b^	88.60 ± 0.23 ^a^	42.99 ± 0.19 ^d^	19.41 ± 0.25 ^c^
SC-ALC	17.05 ± 1.20 ^a^	80.89 ± 0.92 ^d^	52.30 ± 0.76 ^b^	21.00 ± 0.20 ^b^
SC-TRYP	11.85 ± 0.64 ^b^	83.92 ± 0.38 ^c^	50.26 ± 0.21 ^c^	18.78 ± 0.14 ^d^

Different letters within the same column indicate differences between mean values (*p* < 0.05). Values are mean ± SD. PC: Press cake from shrimp heads and shells. No pre-treatment; SC: Press cake pre-treated with SFCE; ALC: Alcalase; TRYP: Trypsin.

**Table 2:** Total amino acid profile of shrimp shell press cakes before and after SFE and their enzymatic hydrolysates (mg/g sample).

	AAs *	PC-300	SC-300	SC-ALC	SC-TRYP	PC-ALC	PC-TRYP
**Hydrophobic Amino Acids**	**TRP**	16.29 ± 1.55	16.20 ± 2.01	8.71 ± 0.25	18.79 ± 0.43	41.74 ± 3.81	33.74 ± 1.48
	**PHE**	12.43 ± 0.28	10.38 ± 0.54	48.25 ± 17.43	58.78 ± 0.79	41.73 ± 3.72	32.45 ± 1.00
	**LEU**	12.22 ± 1.68	8.14 ± 0.79	47.04 ± 7.35	56.65 ± 0.67	70.00 ± 5.64	58.63 ± 1.11
	**ILE**	17.84 ± 1.20	16.32 ± 1.12	33.29 ± 8.26	44.85 ± 7.69	50.10 ± 5.00	40.71 ± 0.72
	**MET**	7.24 ± 0.91	6.28 ± 0.55	14.21 ± 0.03	22.12 ± 1.09	23.35 ± 2.46	18.21 ± 0.40
	**VAL**	18.05 ± 1.01	18.76 ± 1.82	44.70 ± 8.04	53.61 ± 0.69	53.80 ± 5.64	44.86 ± 1.60
	**TYR**	16.20 ± 0.26	16.29 ± 1.55	32.53 ± 1.61	38.69 ± 0.21	41.74 ± 3.81	33.74 ± 1.48
	**ALA**	27.02 ± 1.73	26.12 ± 1.81	89.93 ± 0.95	52.90 ± 2.65	65.25 ± 4.28	55.84 ± 1.17
	**PRO**	16.84 ± 1.49	16.82 ± 1.28	39.23 ± 0.18	36.12 ± 4.02	42.38 ± 2.02	38.15 ± 0.40
**Hydrophilic Amino Acids**	**THR**	28.50 ± 2.36	28.16 ± 1.56	58.24 ± 1.24	68.60 ± 1.99	77.06 ± 5.68	66.14 ± 1.02
	**HIS**	23.75 ± 0.99	26.46 ± 0.43	73.57 ± 4.43	55.71 ± 0.53	68.69 ± 4.43	68.17 ± 4.38
	**LYS**	20.60 ± 1.30	19.52 ± 2.37	68.98 ± 1.70	60.97 ± 1.01	69.46 ± 12.35	63.38 ± 1.55
	**ARG**	31.21 ± 3.81	35.77 ± 3.88	75.22 ± 2.50	74.06 ± 0.13	72.81 ± 6.13	68.26 ± 10.13
	**SER**	21.62 ± 0.54	20.63 ± 4.72	47.66 ± 0.96	58.56 ± 4.38	52.69 ± 5.04	46.75 ± 0.09
	**HYP**	0.29 ± 0.06	0.20 ± 0.01	1.97 ± 0.07	3.43 ± 0.19	0.97 ± 0.07	1.39 ± 0.17
	**GLY**	9.87 ± 0.88	9.59 ± 0.58	49.90 ± 0.07	40.54 ± 0.70	26.91 ± 1.48	22.67 ± 0.01
	**GLU**	79.08 ± 9.21	91.94 ± 16.45	166.63 ± 1.84	143.23 ± 3.63	215.99 ± 3.24	192.34 ± 4.64
	**ASP**	2.08 ± 0.90	7.29 ± 1.52	79.98 ± 1.78	77.16 ± 0.38	48.51 ± 36.30	39.32 ± 2.36
	**C-C**	0.91 ± 0.55	1.74 ± 0.73	2.07 ± 0.01	3.67 ± 0.70	5.33 ± 0.88	2.10 ± 0.87
	SUM	966.80 ± 32.72	833.90 ± 5.06	984.10 ± 13.67	968.44 ± 32.33	362.04 ± 30.71	376.61 ± 43.72

* Amino acid abbreviations: TRP: Tryptophan, PHE: Phenylalanine, LEU: Leucine, ILE: Isoleucine, MET: Methionine, VAL: Valine, TYR: Tyrosine, ALA: Alanine, PRO: Proline, THR: Threonine, HIS: Histidine, LYS: Lysine, ARG: Arginine, SER: Serine, HYP: Hydroxyproline, GLY: Glycine, GLU: Glutamic acid, ASP: Aspartic acid, C-C: Cystine.

**Table 3:** ζ-potential and droplet size measurements of emulsions stabilized by shrimp shell hydrolysates (SPHs) before and after supercritical fluid extraction (SFE), as well as Na-Cas (positive control).

Emulsion	Size								ζ-Potential (mV) 0.2% Protein
	0.2% Peptide				0.4% Peptide				
	D (4,3) (μm)		D (3,2) (μm)		D (4,3) (μm)		D (3,2) (μm)		
	Day 1	Day 8	Day 1	Day 8	Day 1	Day 8	Day 1	Day 8	Day 1
PC-ALC	55.0 ± 2.5 ^b^	71.0 ± 0.3 ^a^	13.0 ± 1.3 ^b^	18.0 ± 1.8 ^c^	28.7±5.1 ^b^	43.8 ± 4.4 ^a^	7.0 ± 0.1 ^a^	18.0 ± 3.0 ^a^	−25.1±7.2 ^a^
PC-TRYP	23.0 ± 2.8 ^c^	28.0 ± 6.6 ^c^	5.0 ± 1.3 ^c^	42.0 ± 3.3 ^a^	18.1 ± 2.3 ^c^	45.1 ± 3.4 ^a^	1.6 ± 0.1 ^b^	7.1 ± 3.9 ^b^	−38.6 ± 0.0 ^b^
SC-ALC	67.0 ± 0.1 ^a^	69.0 ± 3.9 ^a^	21.0 ± 3.4 ^a^	27.0 ± 1.8^b^	35.8 ± 3.2 ^a^	50.8 ± 3.2^a^	1.5 ± 0.3 ^b,c^	24.7 ± 2.4 ^a^	−47.2 ± 4.6 ^c^
SC-TRYP	23.0 ± 0.7 ^c^	54.0 ± 1.1 ^b^	0.7 ± 0.0c ^d^	8.5 ± 2.6 ^d^	10.3 ± 0.1 ^d^	32.8 ± 2.1^b^	1.2 ± 0.0^c^	8.9 ± 6 ^b^	−49.9 ± 3.5 ^c^
Na-Cas	0.37 ± 0.0 ^d^	2.18 ± 0.0 ^d^	0.18 ± 0.0 ^d^	0.20 ± 0.0 ^e^					−43.7 ± 1.1 ^b,c^

Different letters within the same column indicate differences between mean values (*p* < 0.05). Values are mean ± SD.

## Data Availability

The original data presented in the study are included in the article/Supplementary Material; further inquiries can be directed to the corresponding author.

## Supplementary Materials

The following supporting information can be downloaded at: https://www.mdpi.com/article/10.3390/md23030122/s1, Figure S1: The normalized version of Intrinsic fluorescence of SPHs before and after SFE. (—PC-ALC —SC-ALC, —PC-TRYP —SC-TRYP). Figure S2: Surface hydrophobicity (H0) of SC and PC hydrolysates. Different letters within the same column indicate differences between mean values (*p* < 0.05). Values are mean ± SD. (—PC-ALC —SC-ALC, —PC-TRYP —SC-TRYP).
