# Supplementary material for: Influence of Supercritical Fluid Extraction Process on Techno-Functionality of Enzymatically Derived Peptides from Filter-Pressed Shrimp Waste
Source: Mar Drugs. 2025 Mar 11;23(3):122. doi: 10.3390/md23030122 (PMC11943989; doi:10.3390/md23030122)
Supplement: Supplementary file 1 [file marinedrugs-23-00122-s001.zip › marinedrugs-3476353-supplementary.pdf]

Supplementary file S1

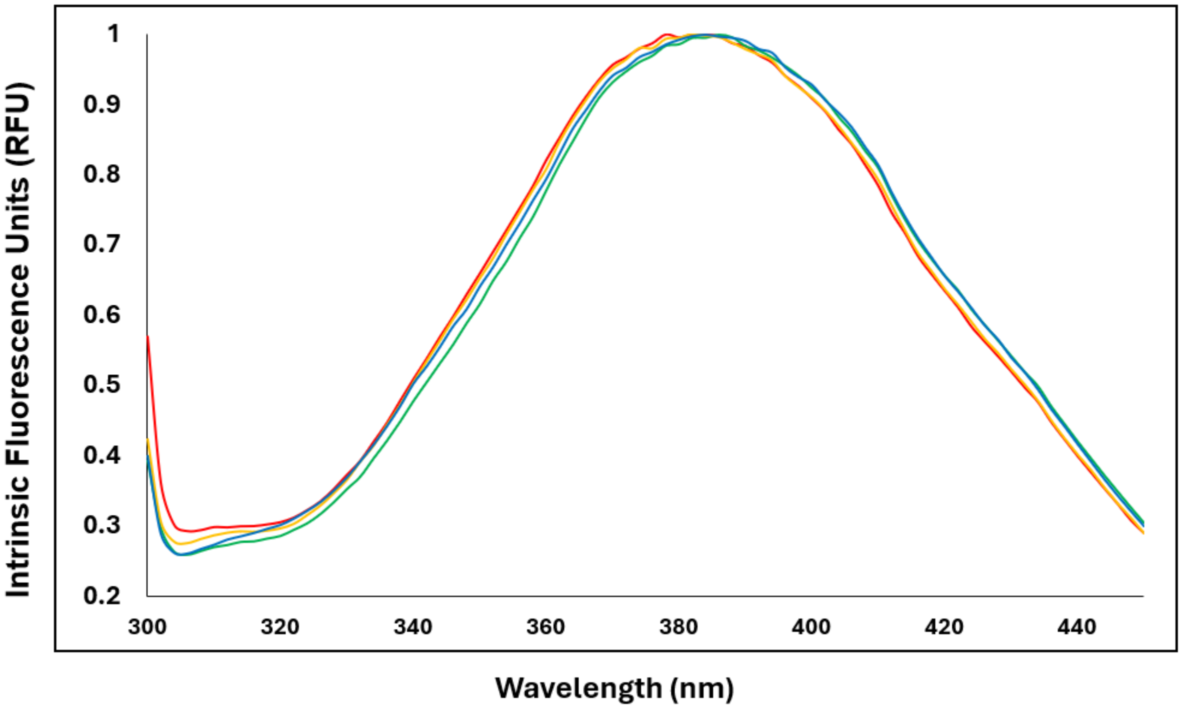

Figure S1. The normalized version of Intrinsic fluorescence of SPHs before and after SFE. (—PC-ALC —SC-ALC, — PC-TRYP — SC-TRYP).
