# Supplementary material for: Influence of Supercritical Fluid Extraction Process on Techno-Functionality of Enzymatically Derived Peptides from Filter-Pressed Shrimp Waste
Source: Mar Drugs. 2025 Mar 11;23(3):122. doi: 10.3390/md23030122 (PMC11943989; doi:10.3390/md23030122)
Supplement: Supplementary file 1 [file marinedrugs-23-00122-s001.zip › marinedrugs-3476353-Supplementary_File_S2.pdf]

Supplementary file S2

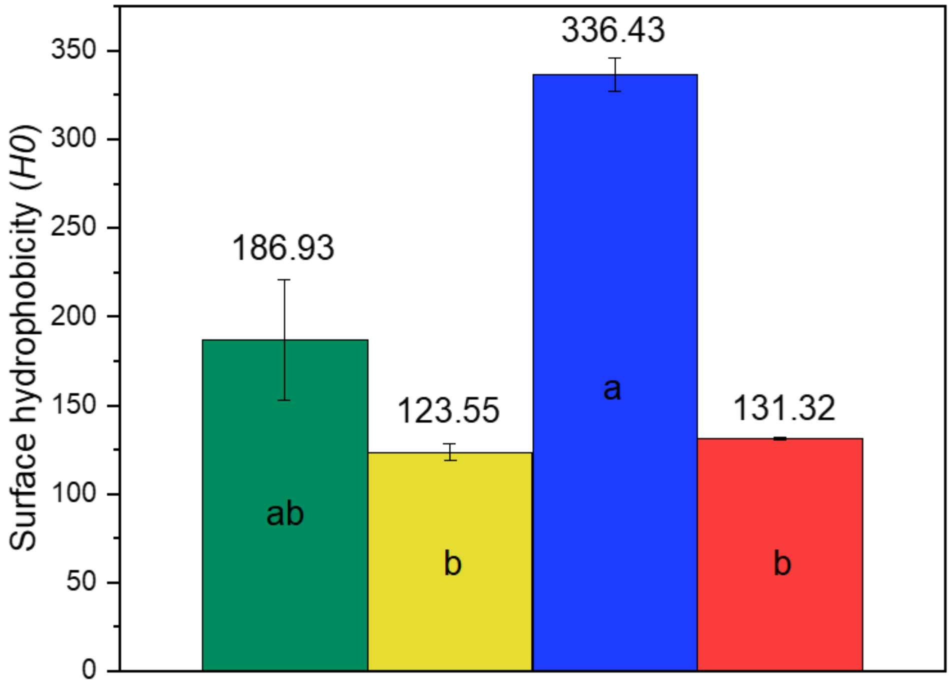

Fig S2. Surface hydrophobicity (H0) of SC and PC hydrolysates. Different letters within the same column indicate differences between mean values ($p < 0.05$). Values are mean ± SD. (—PC-ALC —SC-ALC, — PC-TRYP — SC-TRYP).
